# 

**Published:** 1893-09-30

**Authors:** 


**OUR NEW OFFICES,**
428, Strand, W.C., will henceforth be the Office of this Journal.

**Notices of Births, Deaths, and Marriages** are inserted in this column at a charge of 2s. 6d. for an announcement not exceeding four lines.

### NOTICE TO CORRESPONDENTS.

All MS., Letters, Books for Review, and other matters intended for the Editor should be addressed The Editor, The Lodge, Porchester Square, London, W.
The Editor cannot undertake to return rejected MS., even when accompanied by stamped directed envelope.

**Notice to Advertisers and Subscribers.**

All Advertisements, Orders for Copies of The Hospital, and Business Communications should be addressed to The Publisher, *not to the Editor*, The Hospital, 428, Strand, W.C.
The Cost of Subscription, post free, is as follows:—Per week, 2½d.; per quarter, 2s. 6d.; per half-year, 5s.; per annum, 8s. 6d. Foreign:—Per Annum, 10s. 8d.; payable, in all cases, in advance.

**"Burdett's Hospital Annual," 1893.**

Fourth year of publication. 700 pp. Boards, gilt lettering. A most complete guide to British, Colonial, Indian, and American Hospitals, Dispensaries, Nursing and Convalescent Institutions, and Asylums. Price 5s. Published by The Scientific Press (Limited), 428, Strand, W.C.

**NOTICE.—The Index for Vol. XIII. (ending March, 1893) is on sale at the Office of this Journal, price 2½d., post free.**

### SCRAPS AND GLEANINGS.

The result of the collection at Bournemouth on Hospital Saturday proved an excellent one, exceeding the one last year by over £40.

The committee of the Hospital Saturday Fund, in aid of the Derbyshire Royal Infirmary, recently held their annual excursion.

The annual outdoor festival in aid of the Barnsley Beckett Hospital has just been held, at which the collections amounted to £48.

The report of the Plumpton Convalescent Home for Brighton Children shows a considerable adverse balance; the number of children received last year into the institution was 132.

A varied and successful concert has just been given by the residents and visitors at Folkeston, the entertainment being organised on behalf of the funds of the Beach Rocks Convalescent Home.

A Sunday festival in aid of the Pontefract Dispensary took place recently at Ferrybridge. This is a long looked for resuscitation of an old custom, which has fallen out of use during the last few years.

The Dundee Infirmary has just completed a successful year. The infirmary has advanced steadily in every direction, and it is hoped that in time a complete medical school will be attached to the institution.

A new clock has lately been added to the North-Western Hospital at Haverstock Hill. The Asylums Board have found it necessary to extend the existing buildings of the hospital, which will be proceeded with forthwith.

The money collected at Littlehampton by the amalgamated Friendly Societies of that town and district, at their annual Sunday Demonstration amounted to £26, which sum was handed over to the Chichester Infirmary Committee.

The Saturday collection at Aberdeen for the Sick Children's Hospital was carried out under the management of the Court Granite City Ancient Order of Forresters. The total amount collected reached a lower figure than usual.

It was decided at a meeting of the Southampton Town Council tha after October no cases of infectious diseases of patients residing outside the borough should receive admittance at the Infectious Diseases Hospital in the town.

The Federated Trade and Friendly Societies of Doncaster held their third annual demonstration and open air concert on a recent Sunday afternoon. The surplus of the funds, after all expenses were paid, was handed over to the Doncaster General Infirmary and Dispensary.

The Metropolitan Asylums Board recently ordered an examination and consequent repairs to be made on the ship "Castalea," which in 1884 was converted into a floating small pox hospital, and has since received many hundreds of patients from all parts of the metropolis.

Special efforts are to be made this year to make up the deficit at present existing on the accounts of the Batley Cottage Hospital, the income for which has proved insufficient to meet liabilities, owing more particularly to a decrease of £70 in the last Hospital Saturday Collections.

Mr. John Corbett, of Droitwich, has forwarded a donation of £100 towards the building fund of the Foresters' New Convalescent Home at Clent. When the erection of this home was first considered, 20,000 men agreed to subscribe 1s. each, thus ensuring £1,000 towards the expenses of the institution.

At a meeting of the "North-Eastern Counties Friendly Society's Convalescent Home" it was decided that the use of one bed at the Institution could be secured at the rate of £20 a year. The new rule was made provisional on no one person occupying the bed for a longer period than twenty-four weeks.

The working men of Dudley are agitating for a place on the committee of the Guest Hospital and Dudley Infirmary, to which charities they are respectively the largest subscribers. At the neighbouring towns of West Bromwich and Stourbridge the working classes form an important part of the managing bodies of the local hospitals.

Hospital Sunday in Ryde and Cowes bore a successful issue this year. Street collections took place for the first time and realised £140, whilst from other sources £190 was obtained. These large sums were all the more opportune since on the last report of the hospital (the Isle of Wight Infirmary) a deficit of nearly £1,000 was shown.

The Metropolitan Drinking Fountain and Cattle Trough Association, the only society providing free supplies of water for man and beast in the streets of London, requires £2,000 to pay off outstanding liabilities and carry on the work which they are doing, the area of operations, comprising a district of more than 800 square miles.

A highly successful fête in aid of the Huddersfield Infirmary was organised by the Friendly and Trade Societies of the town. The members of the friendly societies, in full regalia, and the trades showing examples of their works, paraded to the park, where a varied entertainment was held during the afternoon and evening.

The first Hospital Sunday held in Stowmarket, took place recently under the management of the united Friendly Societies of the town. A service was held in the parish church, the collection at which, as also those made in the streets (£11), were handed over towards the funds of the East Suffolk Hospital and Felixstowe Convalescent Home.

The Totnes Cottage Hospital Committee report a satisfactory year's work. The total number of patients admitted during 1892-93 was 34, the total receipts amounted to £215 18s. 1d., and the expenditure to £204 8s. 10d. A site has been presented by Mr. A. M. Champernowne on which a new hospital is to be erected in memory of his late father.

The second annual meeting of the Wellington and District Cottage Hospital was held recently. Since no financial report was presented at the last meeting, the balance sheet included all expenses dating from the opening of the hospital, which reached the sum of £309. The number of patients treated in the hospital was stated to be 63, 85 of which were surgical ones.

The united Friendly Societies of Exeter and Teignmouth respectively held their Hospital Saturday and Sunday parades on succeeding days. The collection at the former town in aid of the Devon and Exeter Hospital reached £31 in all, whilst at Teignmouth the collection reached a higher figure, which was handed over to the Teignmouth Infirmary and Convalescent Home.

Westminster Review.

### Books Received.

"The Value of Hypnotism." By Thos. Crisfield, 34, Southwick Street, W.

J. and A. Churchill.

"Guy's Hospital Reports," Vol. XLIX. Edited by W. Hale White, M.D., and W. H. A. Jacobson, M.A.

Ward, Lock, Bowden, and Co.

"Women Writers: Their Works and Ways." By Catherine J. Hamilton.

John Heywood.

"On Sewage Treatment and Disposal." By Thomas Wardle.

John Wright and Co., Bristol.

"The Clinical Use of Prisms." By Ernest E. Maddox, M.D.
"Sciatic Neuritis." By Robert Simpson, F.R.C.P., L.R.C.S.

Longmans and Co.

"The Refugees." By A. Conan Doyle.

"A Handbook for Mothers." By Jane H. Walker, L.R.C.P. Price 2s. 6d.

Cassell and Co.

"A Manual of Medical Treatment, or Clinical Therapeutics." By J. Burny Yeo, M.D., F.R.C.P.

**Magazines and Pamphlets.**—The Therapeutic Gazette, The Engineering Record, Public Opinion, Medical Record, The Cornhill, International Journal of Surgery, The Sun, Service for the King, Pharmaceutical Journal, The Zenana, Newsagent and Booksellers' Review, The Clinical Journal, Friendly Greetings, The Cottager and Artisan, Light in the Home, Child's Companion, Our Little Boys, Mrs. Heman's Excellent Women Series, The Problem of Human Suffering, The Westminster Review, Literary World, Homœopathic World, Medical Chronicle, Medical Pioneer, Child's Guardian, Provincial Medical Journal, Toilers of the Deep, London, Children's League of Pity Paper, Work and Leisure, Publisher's Circular, Public Health, Homœopathic Review, Charity Organisation Review, The Scalpel, The National Review, The Rural World, Athenæum, The Academy, Journal d'Hygiene, Le Progrès Médical, Journal de Médicine, L'Assistance, Public Opinion (New York), L'Actualité Médicale, Invention, Cassell's Family Magazine, The Picture Magazine, Strand, The Million, Handbook of Western Australia, Edinburgh Medical Journal, Religious Review of Reviews, Indian Medico-Chirurgical Review, Review of the Churches, Reich's Medicinal Anzeiger, Christian World, Amateur Gardening, American Medico-Surgical Bulletin, The Therapist, The Lady's World, The Westminster Budget.

### MEDICAL VACANCIES AND APPOINTMENTS.

**APPOINTMENTS.**

H. J. Alford, M.D.Lond., M.R.C.S.Eng., reappointed Medical Officer of Health for Taunton. C. Averill, M.B., B.S., M.R.C.S., L.S.A., appointed Honorary Surgeon to the Macclesfield Infirmary. E. G. Barnes, M.D.Lond., M.R.C.S.Eng., reappointed Medical Officer of Health for the Hartismere Rural Sanitary District. W. H. Barr, L.R.C.P., L.R.C.S.Edin., reappointed Medical Officer of Health for Bury. H. R. Bramwell, M.B.Edin., M.R.C.S., appointed Honorary Surgeon to the Prudhoe Memorial Convalescent Home, Whitley, Northumberland. W. Calwell, M.A.Q.U.I., M.D.R.U.I., appointed Registrar to the Staff of the Royal Hospital, Belfast. Dr. E. S. Cardell, appointed Medical Officer for the Tonbridge District of the Tonbridge Union. W. H. Clarke, L.R.C.P., L.R.C.S., L.M., appointed Honorary Surgeon to the Macclesfield Infirmary. H. Coates, L.R.C.P. Lond., M.R.C.S.Eng., reappointed Honorary Surgeon to the Salisbury General Infirmary. R. F. Cox, L.R.C P.Lond., M.R.C.S.Eng., appointed Medical Officer of the Kintbury District of the Hungerford Union. J. Davison, M.D.Q.U.I., M.R.C.P.Lond., appointed Honorary Physician to the Royal Victoria Hospital, Bournemouth. B. Dayman, L.R.C.P.Lond., M.R.C.S.Eng., appointed Medical Officer for the Fourth (Nursling) District of the Ramsey Union. G. P. Doyle, M.B., C.M.Aberd., appointed Medical Officer for the No. 8 District of the Newmarket Union. G. H. Fosbroke, M.R.C.S.Eng., D.P.H.Camb., reappointed Medical Officer for the Worcestershire Division of the Evesham Union. C. J. Fuller, L.R.C.P.Lond., M.R.C.S. Eng., appointed Medical Officer for the East Woolwich District of the Woolwich Union. B. C. Gowing, M.R.C.S.Eng., L.S.A., reappointed Medical Officer of Health for the Penistone Urban Sanitary District. H. D. Hey, M.R.C.S., L.R.C.P.Lond., appointed Medical Officer for the Buckland District of the Faringdon Union. G. H. Hooper, M.R.C.S., L.R.C.P., appointed Resident Obstetrical Officer at Charing Cross Hospital. R. W. Jephcott, L.R.C.P., L.M., L.R.C.S.Edin., reappointed Medical Officer for the Alcester District of the Alcester Union. C. G. Jones, L.R.C.P.Edin., M.R.C.S.Eng., appointed Medical Officer and Public Vaccinator for the No. 1 District of the Cardigan Union. J. Kelland, M.B., C.M.Edin., reappointed Honorary Physician to the Salisbury General Infirmary. E. Kingscote, M.B., C.M., L.R.C.S.Edin., reappointed Honorary Surgeon to the Salisbury General Infirmary. S. C. Lawrence, L.R.C.P.Lond., M.R.C.S., appointed Resident Medical Officer at Tilt Cove, Newfoundland. F. F. Lee, M.B.Lond., F.R.C.S. Eng., reappointed Honorary Physician to the Salisbury General Infirmary. L. S. Luckham, M.R.C.S.Eng., L.S.A., reappointed Honorary Surgeon to the Salisbury General Infirmary. F. B. Mallett, M.D., L.R.C.S.Edin., appointed Honorary Consulting Physician to the Bolton Infirmary. C. R. Marshall, M.B., Ch.B.Vic., appointed House Physician to the Royal Infirmary, Manchester. E. Morse, L.R.C.P., L.R.C.S.Edin., reappointed Medical Officer of Health for Great Torrington. D. S. Park, L.R.C.P., F.R.C.S.Edin., reappointed Medical Officer of Health for the Houghton-le-Spring Urban Sanitary District. A. Pern, L.R.C.P.Lond., F.R.C.S.Eng., D.P.H.Camb., reappointed Medical Officer of Health to the South Stonham Rural Sanitary Authority. E. J. Pritchard, M.B., C.M.Glas., reappointed Medical Officer for the Stoke Golding Higham District of the Hinckley Union. T. H. S. Pullin, M.D.St.And., L.R.C.S.Edin., M.R.C.S.Eng., reappointed Medical Officer of Health for the Sidmouth Urban Sanitary District. N. R. J. Rainier, M.R.C.S., L.R.C.P., appointed House Surgeon at Charing Cross Hospital. A. Ramage, L.R.C.P., L.R.C.S.Edin., appointed Medical Officer of Health for Unst. J. Roger, L.R.C.P., L.R.C.S.Edin., L.F.P.S.Glas., appointed Acting Physician to the Larkhall Fever Hospital. T. E. Sandall, B.A. Cantab., M.R.C.S., L.R.C.P., appointed House Physician at Charing Cross Hospital. F. C. Scotson, M.R.C.S.Eng., L.R.C.P. Lond., appointed House Surgeon to the Royal Infirmary, Manchester. H. Skipworth, L.R.C.P.I., M.R.C.S.Eng., reappointed Medical Officer of Health for the Quorndon Urban Sanitary District. J. W. Smith, M.B., C.M.Edin., F.R.C.S.Eng., reappointed Resident Surgical Officer to the Royal Infirmary, Manchester. R. B. Smith, L.R.C.P.Edin., L.R.C.S.I., reappointed Medical Officer for the Wolvey District of the Hinckley Union. J. Steele, reappointed Medical Officer of Health to the Kidsgrove Local Board. R. S. Steele, M.B., C.M. Glas., appointed Medical Officer for the Church Eaton District of the Cannock Union. C. Stein, M.D.Edin., appointed Medical Officer for the Workhouse of the Shipston-on-Stour Union.

### The Hospital Advertiser.

VICTORIA UNIVERSITY.

THE

**YORKSHIRE COLLEGE, LEEDS.**

**UNIVERSITY COLLEGE, BRISTOL.**

J. S. Tew, M.D.Durh., M.B., B.S., reappointed Medical Officer of Health for the Basford Rural Sanitary District. G. W. Till, L.R.C.P., L.M.Edin., L.F.P.S.Glasg., appointed Medical Officer for the Third District of the Worcester Union. H. L. E. Wilks, M.R.C.S.Eng., L.R.C.P.Lond., reappointed House Surgeon to the Salisbury General Infirmary. F. Wilson, L.R.C.P. Edin., M.R.C.S.Eng., appointed Medical Officer of Health for Ramsey. H. Wiskeu, L.F.P.S.Edin., L.F.P.S.Glasg., reappointed Medical Officer of Health for Heywood Borough. G. T. Woods, L.R.C.P., L.R.C.S.Edin., L.F.P.S.Glasg., appointed Medical Officer for the Ripley District of the Knaresborough Union. Mr. A. Wylie, appointed Medical Officer for the Marsden District of the Huddersfield Union.

### VACANCIES.

The following vacancies are announced this week, the amount of salary in each case being given after the name of the institution:

### Resident Medical Officers.

Bethlem Hospital. Two Resident Clinical Students, doubly qualified. Apartments, rations, and attendance provided. Applications, endorsed "Clinical Assistantship," to the Treasurer, at the Bethlem Hospital, by October 2nd.

Belgrave Hospital for Children, 79, Gloucester Street, S.W. House Surgeon. Board and lodging provided. Applications to the Honorary Secretary by September 30th.

East London Hospital for Children, Glamis Road, Shadwell, E. House Physician. No salary. Board and lodging provided. Applications to the Secretary by October 5th.

Leith Hospital. House Physician, House Surgeon, and Surgeon for the Out-patient Department. Appointments for six months. Salary attached to each office is at the rate of £50 per annum, with board in the hospital. Applications to the Secretary, Mr. George V. Mann, 33, Bernard Street, Leith, by September 30th.

Manchester Clinical Hospital for Women and Children, Park Place, Cheetham Hill Road. House Surgeon. Salary £80 per annum, with apartments and board. And Honorary Assistant Surgeon. Applications to Mr. Hubert Teague, Secretary, 38, Barton Arcade, Manchester, by September 30th.

Warneford Hospital, Leamington Spa. House Surgeon, unmarried; doubly qualified. Salary £100 per annum, with board, lodging, and washing. Applications to J. Warren, Secretary, by September 30th.

### Non-Resident Medical Officer.

St. Mungo's College, Glasgow. Chair of Chemistry. Applications, with thirty copies of testimonials, to the Secretary, H. Lamond, by October 2nd.

### PASS LISTS.

### Examining Board in England by the Royal Colleges of Physicians and Surgeons.

The following gentlemen passed the first examination of the Board, under the "Five Years' Regulations," in the subjects indicated:—

PASSED IN ELEMENTARY BIOLOGY.—H. J. Aldous, King's College; F. G. Aldrich, Charing Cross; A. W. Aldridge, Mason College, Birmingham; B. E. G. Bailey, St. Bartholomew's; P. C. Barham, St. Bartholomew's; M. R. Barker, St. Mary's; J. A. Barnes, St. Thomas's; F. Barton, University College, Liverpool; A. St. J. Bateman, King's College; C. L. Batteson, London; F. F. Bond, Westminster; C. H. S. Brown, St. Thomas's; R. Bryan-Haymes, St. Thomas's; E. P. Burns, Mason College, Birmingham; T. W. Byrne, University College, Liverpool; C. S. Cato, Westminster; H. J. Chater, St. Mary's; S. H. G. Cory, St. Mary's; A. Croneen, Guy's; R. I. Cuming, St. Thomas's; T. H. Davies, Middlesex; H. O. P. de Mirimonde, Yorkshire College, Leeds; H. H. J. Edwards, St. Thomas's; M. H. G. Fell, St. Bartholomew's; R. Fell, Guy's; C. E. Fenn, King's College; R. J. de G. Ferguson, University College; W. R. Flint, St. Mary's; H. W. Fox, Guy's; C. H. Francis-Williams, St. George's; J. G. Glasgow, St. Thomas's; A. S. Good, St. George's; G. Hardwick, Yorkshire College, Leeds; J. H. Hare, St. Thomas's; G. W. Harrison, St. Thomas's; R. S. F. Hearn, St. Bartholomew's; C. E. Hogan, St. Bartholomew's; J. Holme, Yorkshire College, Leeds; W. St. A. F. Hubbard, Charing Cross; W. T. Jackson, Owens College, Manchester; W. James, St. Thomas's; E. G. Kent, St. Bartholomew's; E. H. Kitchin, Yorkshire College, Leeds; A. G. Leverton, St. Bartholomew's; J. E. Long, University College, Bristol; C. A. Lower, Guy's; M. R. Maher, University College, Liverpool; W. S. Maugham, St. Thomas's; C. G. Meade, St. Bartholomew's; W. Meade, St. George's; A. Mercer, University College; A. A. Miller, Guy's; S. L. C. Mondy, University College; R. G. Murray, St. George's; A. H. Norris, Owens College, Manchester; B. G. Patch, St. Thomas's; W. D'U. Pemberton, St. Thomas's; H. J. Phillips, St. Thomas's; H. J. Pickering, St. Bartholomew's; R. M. Richards, St. Mary's; O. H. Rogerson, Yorkshire College, Leeds; E. H. Ross, St. Thomas's; W. J. Schuller, London; A. F. Seacome, University College, Liverpool; E. B. Stevenson, St. Bartholomew's; J. L. V. Shortland, King's College; E. Symes, University College, Bristol; A. J. Tayler, King's College; H. W. B. Walling, Guy's; J. H. Watson, University College, Liverpool; R. Willcox, University College, Bristol; B. F. Wingate, St. Mary's; G. Winn, London; R. Le G. Worsley, St. George's.

